# Surgical Management of Gallstone Ileus: Approach, Outcome, Case Report, and Literature Review

**DOI:** 10.7759/cureus.69930

**Published:** 2024-09-22

**Authors:** Madison A Parker, Nathan Kragh, Jeannette Sandoval, Sana Erabti, Basem Soliman

**Affiliations:** 1 Department of Surgery, Texas Tech University Health Sciences Center, Amarillo, USA

**Keywords:** fistula, gallbladder, gallstone ileus, gallstones, general surgery, laparotomy

## Abstract

Gallstone ileus (GSI) is a condition caused by migrating gallstones lodged in the terminal ileum. Stone migration results from fistula formation, typically between the gallbladder and duodenum, termed a cholecystoduodenal fistula. This mechanical obstruction has high mortality and requires prompt intervention. This discussion focuses on two GSI cases caused by cholecystoduodenal fistula managed by the surgical removal of the mechanical obstruction and a conservative approach to the fistula. Patient 1 is a 51-year-old male with no significant past medical history. After radiological imaging and laboratory findings raised concern for cholecystoduodenal fistula, the patient underwent a diagnostic laparoscopy with small bowel resection. The second patient is a 74-year-old female with a past medical history of hypertension, myocardial infarction, and laparoscopic uterine lift. The patient underwent diagnostic laparoscopy, lysis of adhesions, mini-laparotomy, and segmental small bowel resection with primary anastomosis. Many factors lead to gallstone formation, including gender, genetics, ethnicity, etc. Fistulas are formed from chronic inflammation and tissue necrosis from gallstone pressure on surrounding tissue. Classic radiologic findings of GSI are pneumobilia, bowel obstruction, and gallstones outside the gallbladder. The most common approach to GSI management is solely relieving the mechanical obstruction by an enterotomy proximal to the obstruction, associated with reduced mortality. Both patients had good outcomes which we attributed to our surgical removal of the small bowel obstruction and a non-operative approach to the cholecystoduodenal fistula.

## Introduction

This case study presents two cases of gallstone ileus (GSI), managed with surgical intervention, with positive outcomes. GSI is a rare condition caused by migrating gallstones that are typically lodged in the terminal ileum [1,2]. Stone migration is a result of a fistula, typically between the gallbladder and the duodenum, termed a cholecystoduodenal fistula [1]. A cholecystocolic fistula is a less common fistula between the gallbladder and the large intestine and can result in a gallstone sigmoid ileus [1]. Gallbladder fistulas are rare, accounting for 0.9% of biliary tract surgeries, of which 70% are cholecystoduodenal and 10-20% cholecystocolic [1]. This type of non-strangulated mechanical obstruction has a high mortality of 12-17% and requires prompt management [2]. This discussion will focus on GSI caused by cholecystoduodenal fistula and a surgical approach to address only the mechanical obstruction combined with a conservative approach to the fistula.

## Case presentation

Case 1

**Figure 1:**
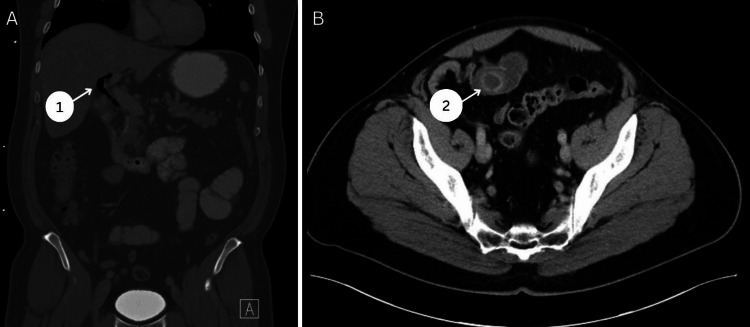
Preoperative CT images for patient 1 in the coronal and axial views 1: pneumobilia in the common bile duct; 2: 2.4 cm gallstone in the distal ileum CT: computed tomography

The patient is a 51-year-old gentleman with no chronic health issues or previous surgeries with a three-day history of epigastric pain and associated nausea, vomiting, and intermittent fevers. He presented to the local emergency department and obtained laboratory and radiological studies as per emergency department staff. See Table 1 for significant laboratory results. All other labs were unremarkable with no leukocytosis. Radiological imaging revealed pneumobilia with air in the common bile duct and gallbladder lumen raising concern about cholecystoduodenal fistula (see Figure 1A). The distal ileum had a 2.4 cm density with significant dilation proximally consistent with GSI (see Figure 1B). A physical exam revealed abdominal distention, hypoactive bowel sounds, and mild tenderness to the epigastric area. The patient was taken promptly to the operating room and underwent a diagnostic laparoscopy, mini-laparotomy, and segmental small bowel resection containing a large gallstone at the distal ileum. Significant small bowel distention from the duodenum to the distal ileum increased the complexity of the case. The patient was returned to the surgical floor where he participated in therapeutic activities and recovered well. He was discharged on postoperative day 5. The patient was seen in the surgery clinic two weeks and three months after discharge and had no further complaints or issues. Cholecystoduodenal fistula was discussed with the patient, and since he is asymptomatic, there is no surgical intervention planned. 

**Table 1:** Significant laboratory values for patient 1 ALT: alanine transaminase; AST: aspartate aminotransferase

	Patient values	Reference ranges
Total bilirubin (mg/dL)	1.8	0.1-1.2
Phosphorus (mg/dL)	74	2.5-4.5
ALT (U/L)	49	4-36
AST (U/L)	64	8-33

Case 2

**Figure 2:**
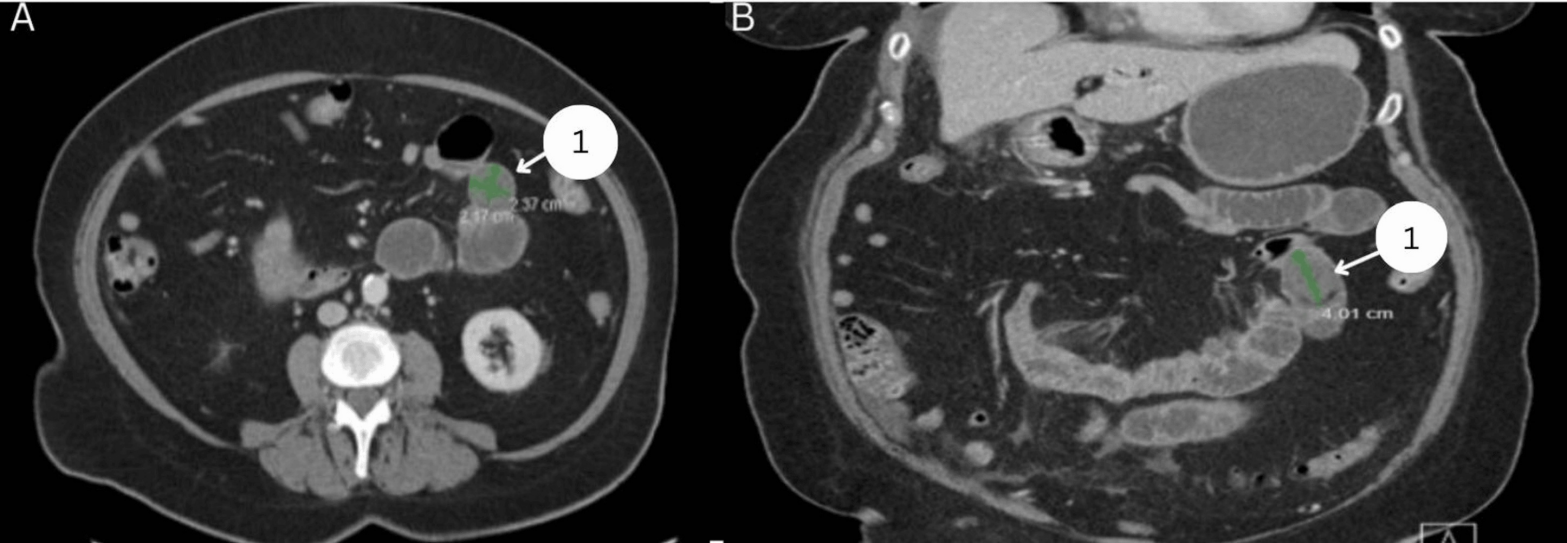
Preoperative CT images for patient 2 in the axial and coronal views 1: 4 cm gallstone in the loop of the jejunum CT: computed tomography

The patient is a 74-year-old female with a past medical history significant for hypertension, myocardial infarction, and surgical history of laparoscopic uterine lift. She presented to the local hospital with complaints of severe epigastric and right upper quadrant pain for one week with associated chills, nausea, vomiting, PO (by mouth) intolerance, and constipation. On presentation, the patient was evaluated by laboratory and radiological studies. See Table 2 for significant laboratory results. Other laboratory studies were unremarkable. Computed tomography (CT) of the abdomen and pelvis with intravenous (IV) contrast was obtained noting a decompressed gallbladder with a markedly thickened and enhancing wall and a fistulous connection between the lateral gallbladder wall and the second portion of the duodenum with associated pneumobilia. A 4 cm lamellated structure was seen within a loop of jejunum in the left mid-abdomen, consistent with an impacted gallstone (see Figure 2A, 2B). Small bowel immediately distal to the presumed gallstone demonstrated wall thickening mucosal enhancement inflammatory changes, consistent with infectious, inflammatory, or reactive enteritis. Additionally, there was a transition to decompressed ileum at this bowel segment, consistent with GSI. A physical exam revealed significant tenderness to palpation in the right upper quadrant and epigastric region. The patient was taken urgently to the operating room for diagnostic laparoscopic, lysis of adhesions, mini-laparotomy, and segmental small bowel resection with primary anastomosis. A transition point was found in the mid-jejunum with palpable intraluminal stone, and the segment of the bowel surrounding the impacted stone was dusky with punctate hemorrhage and thus was resected. The patient was returned to the surgical ward for postoperative care. She was discharged from the hospital on postoperative day 5. The patient was seen in the surgery clinic two weeks and three months after discharge without further issues or complaints.

**Table 2:** Significant laboratory values for patient 2 WBC: white blood cells

	Patient values	Normal
WBC (cells/mm^3^)	17,500	4500-11,000
Serum Na+ (mmol/L)	132	135-147
Serum Cl- (mmol/L)	96	96-100
Lipase (U/L)	190	10-140

## Discussion

Stone pathophysiology

Gallstones or cholelithiasis is typically formed in the gallbladder but can also develop in the hepatic duct or common bile duct [3]. There are a wide variety of factors that lead to gallstone formation which include gender, genetics, ethnicity, obesity, rapid weight loss, glucose intolerance, insulin resistance, high glycemic intake, diabetes, hypertriglyceridemia, drugs, and pregnancy [3]. Abnormal metabolism of cholesterol, bile salts, and bilirubin leads to stone formation that can be a variety of colors, shapes, and sizes [3].

Fistula pathophysiology

Fistulas are formed from the gallstones putting pressure on the surrounding tissues with chronic inflammation, necrosis, and then fistula creation [4]. Typically, the surrounding tissues are densely adhered due to chronic cholecystitis [5]. Fistulas to the duodenum are the most common followed by the stomach, jejunum, and colon [1,2,4,5]. The fistulas give rise to releasing the gallstones to the lower gastrointestinal (GI) tract and can cause bowel obstruction. Typically, the diameter of the stone needs to be 2-2.5 cm or more to cause issues in susceptible areas where there is a narrowing in the GI tract [4,5]. Other causes of fistulization without cholelithiasis or cholecystitis include peptic ulcer, pancreatic malignancy, Crohn's, ulcerative colitis, diverticulitis, abdominal trauma, or previous surgery [6]. 

Clinical presentation

Early diagnosis is essential and directly related to the high mortality rate of this disease process up to 30% [2,4]. Patients will typically present with symptoms of small bowel obstruction and previous biliary colic, jaundice, or cholecystitis described as the Mordor triad [4]. The rarity of GSI can lead to a delay in diagnosis and typically affects older populations with comorbidities [5]. Clinical presentation can be nonspecific with a variety of symptoms including abdominal pain, distention, bloating, nausea, vomiting, jaundice, and other nonspecific symptoms of small bowel obstruction [5]. Any patients with cholelithiasis and previously mentioned symptoms should have GSI included in the differentials [5]. The wide variety of clinical presentations complicates the clinical picture and leads to delays in diagnosis, increasing mortality and complications. 

Imaging/labs

Imaging includes CT, magnetic resonance imaging (MRI), ultrasound (US), and plain film X-ray [5]. Classic findings of GSI known as the Rigler triad or Rigler sign are pneumobilia, bowel obstruction, and gallstone outside the gallbladder [5]. US has limitations in seeing pneumobilia, and plain film X-ray may not show gallstones as most are not radiopaque [5]. MRIs are time-consuming, require the patient to be still for extended periods, and can delay care. CTs are the most reliable with high sensitivity and specificity [5,6]. Labs may be nonspecific depending on the acuity of the patient [6]. 

Surgical interventions

Several surgical approaches exist and are highly variable depending on the patient's presentation, the surgeon's experience, and the resources available. The most common approach to GSI management is solely relieving the mechanical obstruction by creating an enterotomy proximal to the obstruction to remove the stone, which is associated with reduced mortality [7]. Resolution of the cholecystoduodenal fistula can be performed at a later date if symptomatic resolution is not achieved. If bowel distal to the obstruction is deemed non-viable, a resection with anastomosis may be necessary. A second strategy for GSI management is a two-stage approach consisting of a simple enterolithotomy during the first procedure with a second operation to perform cholecystectomy and biliary fistula repair [7]. A complication of these strategies is GSI recurrence, sometimes as early as one-week post-op [8]. In cases when symptoms recur or persist, a cholecystectomy with fistula repair can be planned for a later date [9]. Recurrence can be avoided with a one-stage approach where a cholecystectomy and fistula repair are performed in the same operation as the small bowel obstruction resolution, but this is associated with higher mortality [7]. For some patients, symptoms resolve and there is never a need for cholecystectomy or fistula repair at all [9].

## Conclusions

GSI is a rare bowel obstruction requiring timely surgical intervention due to its high morbidity and mortality. Because it involves a combination of multiple disease processes (chronic cholecystitis, gallbladder fistula, small bowel obstruction), there are a variety of surgical options for treatment. Taking the conservative route by prioritizing obstruction relief and avoiding a second procedure unless necessary seems to be a favorable option as it reduces mortality and is a simpler approach overall. Because this is an uncommon pathology, identifying the ideal surgical intervention will rely on increasing the number of case studies in the literature. Regardless, each patient's unique presentation and indications should inform the approach taken by the surgical team.
